# An adenine base editor with expanded targeting scope using SpCas9‐NGv1 in rice

**DOI:** 10.1111/pbi.13120

**Published:** 2019-04-30

**Authors:** Katsuya Negishi, Hidetaka Kaya, Kiyomi Abe, Naho Hara, Hiroaki Saika, Seiichi Toki

**Affiliations:** ^1^ Plant Genome Engineering Research Unit Institute of Agrobiological Sciences National Agriculture and Food Research Organization Tsukuba Ibaraki Japan; ^2^ Graduate School of Agriculture Ehime University Matsuyama Ehime Japan; ^3^ Graduate School of Nanobioscience Yokohama City University Yokohama Kanagawa Japan; ^4^ Kihara Institute for Biological Research Yokohama City University Yokohama Kanagawa Japan; ^5^ Present address: Biotherapy Institute of Japan Inc. 1‐18‐2 Sakura Tsukuba Ibaraki 305‐0003 Japan

**Keywords:** adenine base editor, CRISPR/Cas9, genome editing, *Oryza sativa*, protospacer adjacent motif, SpCas9‐NG

Dear Editor,

The CRISPR/Cas9 genome editing system induces mainly small insertions or deletions at the target site. In contrast, base editors are precise genome editing tools that enable base conversion at a target site without inducing a DNA double‐strand break. Two types of base editors have now been developed: cytosine base editors (CBEs)—cytidine deaminase fused with catalytically impaired Cas9—can efficiently convert a C–G base pair to a T–A pair, while adenine base editors (ABEs)—Cas9 nickase (nCas9) plus engineered *Escherichia coli* adenosine deaminase (TadA)—enable A–T to G–C conversions (Gaudelli *et al*., 2017; Kim, 2018). Both CBEs and ABEs have been applied successfully in plants such as *Arabidopsis*, wheat and rice (e.g., Hua *et al*., 2018, 2019; Li *et al*., 2018). However, potential base editing targets remain restricted by the requirement for protospacer adjacent motif (PAM) sequences around these sites. Cas9 derived from *Streptococcus pyogenes* (SpCas9)—currently the Cas9 most widely used for genome editing in many organisms—requires as a PAM an NGG sequence adjacent to the target site. To extend Cas9‐recognized sites, several groups have engineered Cas9 variants with amino acid substitutions and modified PAM sequences. Using these Cas9 variants in base editors could expand the scope of base editing sites (Endo *et al*., 2019; Hu *et al*., 2018; Hua *et al*., 2019; Nishimasu *et al*., 2018). Engineered SpCas9s, SpCas9‐NG variants and xCas9 variants that can recognize NG as PAM have been reported recently (Hu *et al*., 2018; Nishimasu *et al*., 2018). SpCas9‐NG and xCas9 variants can induce mutation at target sites with NG PAMs in rice (Endo *et al*., 2019; Wang *et al*., 2019), while the *in vitro* DNA cleavage activity of SpCas9‐NGv1 (reported as ARVRFRR; Nishimasu *et al*., 2018), which includes a 7‐aa mutation (R1335A/L1111R/D1135V/G1218R/E1219F/A1322R/T1337R) in the PAM‐interacting domain, is higher than that of xCas9 (Endo *et al*., 2019; Nishimasu *et al*., 2018). SpCas9‐NGv1 is also available for C–T base conversions as CBEs in rice (Endo *et al*., 2019). Here, we report the development of novel ABEs using nSpCas9‐NGv1 (ABE7.10‐nSpCas9‐NGv1) and show that SpCas9‐NGv1 can induce A–G substitutions at a target site containing NG as PAM in the rice genome.

We first examined whether the ABE system can function in rice callus. We synthesized an *Arabidopsis thaliana* codon‐optimized coding region containing TadA‐wt‐(XTEN)_2_‐TadA*7.10‐(XTEN)_2_ (Gaudelli *et al*., 2017). Then, *A*. *thaliana* codon‐optimized nSpCas9 (D10A) and 3×NLS were fused to the C‐terminus of the coding region (Figure 1a). We chose four target sites (sgOs‐siteG1: chr05_27537337–27537356; sgOs‐site2: chr05_27536662–27536681; sgOs‐site3: chr02_19223102–19223121; and sgOs‐site4: chr11_23944837–23944856) with NGG at their 3′ end as PAM in the rice genome (Figure 1b, c). A binary vector harbouring a single guide RNA (sgRNA) and ABE7.10‐nSpCas9 expression cassettes was integrated into the rice genome *via Agrobacterium*‐mediated transformation. After culturing for 1 month, genomic DNA extracted from the transformed calli was subjected to cleaved amplified polymorphic sequences (CAPS) analysis. All four target sites have restriction enzyme sites overlapping the editing window of previously reported ABEs (Gaudelli *et al*., 2017; Hua *et al*., 2018). Undigested DNA fragments were detected in transgenic calli, suggesting that ABE7.10‐nSpCas9 had indeed induced the desired mutation, and with roughly the same target window as other reported ABEs (Figure 1b). The ratios of mutated callus detected by CAPS analysis at the four different target sites were 81.7% (76/93 calli: site G1), 63.8% (51/80 calli: site G2), 68.5% (63/92 calli: site G3), and 69.3% (61/88 calli: site G4). DNA sequences around the target sites were confirmed using several transgenic calli in which undigested DNA fragments were clearly detected by CAPS analysis. Sequence analysis revealed that ABE7.10‐nSpCas9 efficiently induced an A–G substitution at a position −16 to −13 nt upstream from the PAM (Figure 1c). This target window is almost the same as that seen in data reported previously in human and rice cells (Gaudelli *et al*., 2017; Hua *et al*., 2018). We detected no substitutions other than from A to G. These results showed that ABE7.10‐nSpCas9 can direct A–G substitutions effectively at NGG PAM target sites.

**Figure 1:**
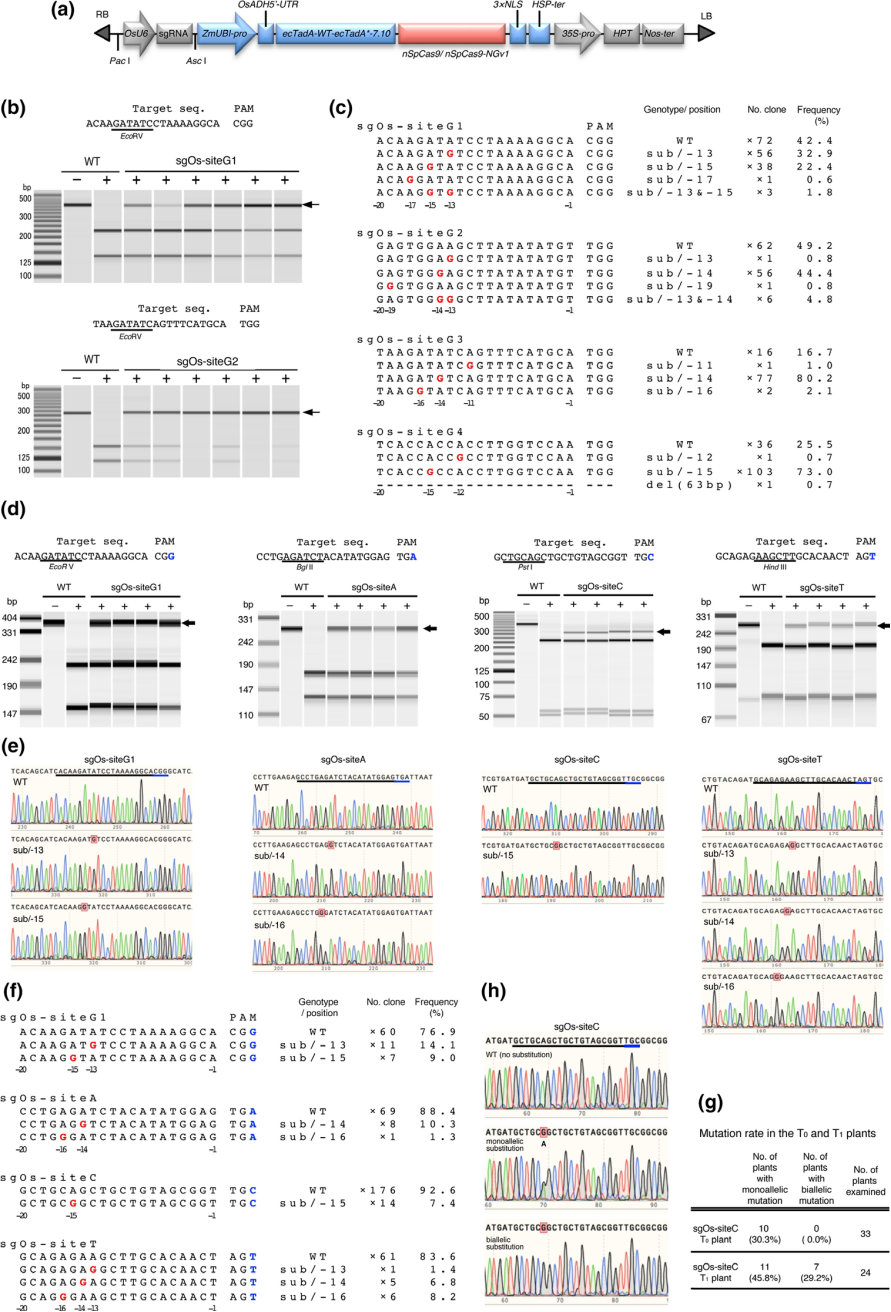
ABE7.10‐nSpCas9‐NGv1 can induce A‐to‐G substitution at target sequences with 5′‐NG‐3′ as PAM. (a) Expression construct for *sgRNA* and *ABE7.10‐nSpCas9* or *ABE7.10‐nSpCas9‐NGv1* (R1335A/L1111R/D1135V/G1218R/E1219F/A1322R/T1337R) in rice. nSpCas9; SpCas9 nickase (D10A). (b) CAPS analysis at target locus with NGG as PAM in ABE7.10‐nSpCas9 transformed rice calli. Arrows indicate undigested fragments. −/+ without/with restriction enzyme. (c) Mutations detected in ABE7.10‐nSpCas9 transformed rice calli. The wild‐type sequence is shown at the top. Substituted bases and deletions are shown in red letters and dashes, respectively. Sequence genotype, position of the mutated base, number of sequenced clones and mutation frequency (%) are noted to the right of each sequence (sub; substitution, −; deletion, ×; number of sequenced clones). Numbers below the sequence indicate the number of bases from the PAM (first letter of PAM is +1). (d) CAPS analysis at target locus with NGG, NGA, NGC and NGT as PAM in ABE7.10‐nSpCas9‐NGv1 transformed rice calli. (e) Sanger sequencing chromatograms of wild type and mutants induced by ABE7.10‐nSpCas9‐NGv1. The black and blue underlining indicates the target sequence and PAM, respectively. Substituted bases are indicated by red squares. (f) Mutations induced in the target sequences. (g) Mutation rate in T_0_ and T_1_ plants. (h) Representative Sanger sequencing chromatogram of T_1_ plants.

The newly engineered SpCas9‐NGv1 can recognize NG as PAM, which increases the target sites available to Cas9‐mediated genome editing (Endo *et al*., 2019; Nishimasu *et al*., 2018). To apply the SpCas9‐NGv1 to the ABE system, we constructed ABE7.10‐nSpCas9‐NGv1 harbouring the nickase type of SpCas9‐NGv1 instead of nSpCas9 (Figure 1a). We chose four target sites (sgOs‐siteG1; sgOs‐siteA: chr06_1770405–1770424; sgOs‐siteC: chr04_33074491–33074510; and sgOs‐siteT: chr06_2926795–2926814) in the rice genome with NGG, NGA, NGC and NGT, respectively, as PAM sequences (Figure 1d). Genomic DNA was extracted from ABE7.10‐nSpCas9‐NGv1 transformed calli. CAPS analysis showed the presence of the undigested band in several transgenic calli (66/88 calli: site G1, 42/88 calli: site A, 10/40 calli: site C, 44/77 calli: site T). We investigated the mutation patterns and efficiency of ABE7.10‐nSpCas9‐NGv1 at each target site by sequence analysis (Figure 1e, f). At the NGA (site A), NGC (site C) and NGT (site T) PAM target sites, A–G substitutions were detected. The substitution induced by ABE7.10‐nSpCas9‐NGv1 was positioned around 13–16 nt upstream of the PAM (Figure 1f). ABE7.10‐nSpCas9‐NGv1 could induce the mutation in a target window similar to that of ABE7.10‐nSpCas9 (Figure 1c, e, f). At the NGG PAM target site (site G1), the substitution efficiency of ABE7.10‐nSpCas9‐NGv1 was lower than that of ABE7.10‐nSpCas9 (Figure 1c, f), consistent with the results of SpCas9‐NGv1‐mediated target mutagenesis (Endo *et al*., 2019). As seen with ABE7.10‐nSpCas9, ABE7.10‐nSpCas9‐NGv1 did not induce any other substitution in rice calli. These results indicate that ABE7.10‐nSpCas9‐NGv1 could expand the scope of A–G base editing sites with NG PAMs in rice callus. We checked the genotypes of 33 independent regenerated plants (T_0_) and detected the monoallelic A–G substitution in 10 plants (30.3%) (Figure 1g). Of 24 T_1_ plants, 7 (29.2%) harboured biallelic and 11 (45.8%) monoallelic A–G substitution mutants (Figure 1g, h). These data demonstrate that ABE7.10‐nSpCas9‐NGv1‐induced A–G substitution was inherited to the next generation in rice.

In this study, we developed new ABEs using SpCas9‐NGv1 that have NG as PAM and successfully induced A–G base substitutions in endogenous sites of the rice genome. Furthermore, the induced mutations were stably inherited to the next generation. We recently also reported targeted mutagenesis and C–T base editing using SpCas9‐NGv1 in plants (Endo *et al*., 2019). These base editing technologies using SpCas9‐NGv1 provide high‐precision molecular engineering tools allowing regulation of enzyme activity, gene expression, protein interaction and protein structure. During preparation of this manuscript, Jeong *et al*. (2019) reported that ABE using SpCas9‐NG converted A to G at endogenous target sites containing NG PAMs in human cells.

## Conflict of interests

All authors declare no conflict of interest.

## References

[pbi13120-bib-0001] Endo, M. , Mikami, M. , Endo, A. , Kaya, H. , Itoh, T. , Nishimasu, H. , Nureki, O. *et al*. (2019) Genome editing in plants by engineered CRISPR–Cas9 recognizing NG PAM. Nat. Plants, 5, 14–17. 30531939 10.1038/s41477-018-0321-8

[pbi13120-bib-0002] Gaudelli, N.M. , Komor, A.C. , Rees, H.A. , Packer, M.S. , Badran, A.H. , Bryson, D.I. and Liu, D.R. (2017) Programmable base editing of A*T to G*C in genomic DNA without DNA cleavage. Nature, 551, 464–471. 29160308 10.1038/nature24644 PMC5726555

[pbi13120-bib-0003] Hu, J.H. , Miller, S.M. , Geurts, M.H. , Tang, W. , Chen, L. , Sun, N. , Zeina, C.M. *et al*. (2018) Evolved Cas9 variants with broad PAM compatibility and high DNA specificity. Nature, 556, 57–63. 29512652 10.1038/nature26155 PMC5951633

[pbi13120-bib-0004] Hua, K. , Tao, X. , Yuan, F. , Wang, D. and Zhu, J.K. (2018) Precise A.T to G.C base editing in the rice genome. Mol. Plant, 11, 627–630. 29476916 10.1016/j.molp.2018.02.007

[pbi13120-bib-0005] Hua, K. , Tao, X. and Zhu, J.K. (2019) Expanding the base editing scope in rice by using Cas9 variants. Plant Biotechnol. J. 17, 499–504. 30051586 10.1111/pbi.12993 PMC6335069

[pbi13120-bib-0006] Jeong, Y.K. , Yu, J. and Bae, S. (2019) Construction of non‐canonical PAM‐targeting adenosine base editors by restriction enzyme‐free DNA cloning using CRISPR‐Cas9. Sci. Rep. 9, 4939. 30894632 10.1038/s41598-019-41356-1 PMC6426851

[pbi13120-bib-0007] Kim, J. (2018) Precision genome engineering through adenine and cytosine base editing. Nat. Plants, 4, 148–151. 29483683 10.1038/s41477-018-0115-z

[pbi13120-bib-0008] Li, C. , Zong, Y. , Wang, Y. , Jin, S. , Zhang, D. , Song, Q. , Zhang, R. *et al*. (2018) Expanded base editing in rice and wheat using a Cas9‐adenosine deaminase fusion. Genome Biol. 19, 59. 29807545 10.1186/s13059-018-1443-z PMC5972399

[pbi13120-bib-0009] Nishimasu, H. , Shi, X. , Ishiguro, S. , Gao, L. , Hirano, S. , Okazaki, S. , Noda, T. *et al*. (2018) Engineered CRISPR‐Cas9 nuclease with expanded targeting space. Science, 361, 1259–1262. 30166441 10.1126/science.aas9129 PMC6368452

[pbi13120-bib-0010] Wang, J. , Meng, X. , Hu, X. , Sun, T. , Li, J. , Wang, K. and Yu, H. (2019) xCas9 expands the scope of genome editing with reduced efficiency in rice. Plant Biotechnol. 17, 709–711. 10.1111/pbi.13053 PMC6419569 30549238

